# Rotator cuff tear healing process with graft augmentation of fascia lata in a rabbit model

**DOI:** 10.1186/s13018-018-0900-4

**Published:** 2018-08-13

**Authors:** Takeshi Kataoka, Takeshi Kokubu, Tomoyuki Muto, Yutaka Mifune, Atsuyuki Inui, Ryosuke Sakata, Hanako Nishimoto, Yoshifumi Harada, Fumiaki Takase, Yasuhiro Ueda, Takashi Kurosawa, Kohei Yamaura, Ryosuke Kuroda

**Affiliations:** Department of Orthopaedic Surgery, Kobe University Graduate School of Medicine, 7-5-1 Kusunoki-cho, Chuo-ku, Kobe, Hyogo 650-0017 Japan

**Keywords:** Large and massive rotator cuff tear, Fascia lata augmentation, Type III collagen, Ultimate failure load

## Abstract

**Background:**

Fascia lata augmentation of massive rotator cuff tears has shown good clinical results. However, its biological effect during the early healing process is not clearly understood. The purpose of the study was to evaluate the biological efficacy of fascia lata augmentation during the early healing process of rotator cuff tears using a rabbit rotator cuff defect model.

**Methods:**

The infraspinatus tendon was resected from the greater tuberosity of a rabbit to create a rotator cuff tear. The tendon edge was directly sutured to the humeral head. The rotator cuff repaired site was augmented with a fascia lata autograft (augmentation group, group A). The rotator cuff defect in the contralateral shoulder was repaired without augmentation (reattachment group, group R). A group with intact rotator cuff was set as the control group. Histological examinations and mechanical analysis were conducted 4 and 8 weeks postoperatively.

**Results:**

In the HE staining, the tendon maturing score of group A was higher than that of group R at 4 weeks postoperatively. In the safranin O staining, proteoglycan staining at the repaired enthesis in group A at 4 weeks postoperatively was stronger than that in group R. Picrosirius red staining showed that type III and type I collagen in group A was more strongly expressed than that in group R at 4 weeks postoperatively. The ultimate failure load of the infraspinatus tendon–humeral head complex in group A was statistically higher than that in group R at 4 weeks postoperatively. The ultimate failure load of group A was similar to that of the control group.

**Conclusion:**

The biological and mechanical contribution of fascia lata augmentation for massive rotator cuff tears was analyzed in this study. Type III collagen was reported to be expressed during the tendon healing process. Although the biological action similar to natural ligament healing occurred around the fascia lata grafts, type III collagen was gradually replaced by type I collagen as the tissue matured. Our results suggest that fascia lata augmentation could stimulate biological healing and provide initial fixation strength of the repaired rotator cuff.

## Background

Rotator cuff tears have been reported to occur in > 50% of patients aged > 60 years [1]. They cause chronic pain and severe dysfunction, leading to degenerative changes in the glenohumeral joint [2, 3]. Excellent outcomes of arthroscopic rotator cuff repair for small and medium tears have been recently reported [4, 5]. In contrast, large and massive rotator cuff tears are challenging for surgeons. Various surgical procedures, such as musculotendinous transfer [6], autograft augmentation [7], or synthetic materials [8] are available for the repair of massive rotator cuff tears. However, retears have been a common complication after surgical repair of such tears. The retear rates have been reported to be 14–66% for large or massive tears [9–13]. Shoulders without cuff retear had better function during daily activities and better range of motion than shoulders with retears [3]. Retears are presumed to result from high tension and insufficient initial biological healing at the repair site [14]. We have performed a single-row repair with graft augmentation of the fascia lata for large and massive rotator cuff tears to reduce tension at the tendon–bone repair site [15]. However, its biological effect during the early healing period has not been clearly understood. The purpose of the study was to evaluate the biological efficacy of fascia lata augmentation during the early healing process of rotator cuff tears using a rabbit rotator cuff defect model.

## Methods

### Animal model of rotator cuff repair

**Fig. 1:**
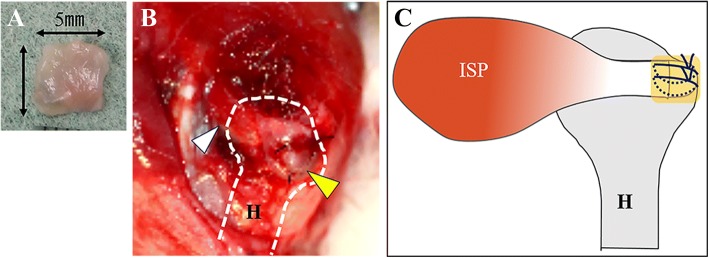
Animal model of rotator cuff repair. **a** Macrography of the fascia lata autograft (5 × 5 mm). **b** Fascia lata autograft was transplanted on the rotator cuff repaired site (white arrow head, infraspinatus tendon; yellow arrow head, transplanted fascia lata; H, humeral head). **c** Scheme of ISP (infraspinatus tendon) repair with fascia lata. Yellow square is transplanted fascia lata

This investigation was approved by the Institutional Animal Care and Use Committee and carried out according to the Kobe University Animal Experimentation Regulations (permission number P140102). Twenty-four skeletally mature female Japanese white rabbits were used in this study. Their age was 16 weeks, and their mean weight was 3.1 kg (range, 2.7–3.5 kg). Intravenous pentobarbital (30 mg/kg; Kyoritsu Seiyaku, Tokyo, Japan) was administered to rabbits. Lidocaine (1%, 10 mg/kg; AstraZeneca, London, UK) was subcutaneously injected. Rabbits were placed in a lateral position, a 3-cm skin incision was made over the lateral border of the acromion on both shoulders, and the infraspinatus tendons were exposed. The infraspinatus tendons (5 × 5 mm) were resected from the greater tuberosity to create rotator cuff defects, and decortication was subsequently performed at the greater tuberosity of the humeral head (1 × 5 mm) to remove normal enthesis and expose the cancellous bone. Two bone tunnels were created from the footprint in the left shoulder using a 1.0-mm Kirschner wire, and tendon edge was sutured directly to the humeral head using a 4-0 nylon suture (reattachment group, group R). Tendons in the right shoulder were repaired similarly, and the repaired site was augmented with a fascia lata autograft (5 × 5 mm) (augmentation group, group A) (Fig. 1). A fascia lata autograft was harvested from the lateral aspect of the right thigh. The fascia lata was transplanted on the repair site and sutured using two simple stitches with a 4-0 nylon. All rabbits were mobilized postoperatively and were immediately allowed to move freely within their cages. The rabbits were euthanized using overdose of pentobarbital sodium at 4 and 8 weeks postoperatively. Four rabbits did not undergo surgery and were examined mechanically as normal controls.

### Histological analysis

The infraspinatus tendon–humeral head complexes were fixed in 4% paraformaldehyde, decalcified with 0.25 mol/l ethylenediaminetetraacetic acid in a phosphate-buffered saline (pH 7.5), and embedded in paraffin. Continuous sections (7 μm in thickness) were cut in the sagittal plane in the middle of the tendons. Tissue sections were stained with hematoxylin–eosin (HE) and safranin O for the histologic characterization of tissue composition, and the histological findings were evaluated at two points: the tendon proper and tendon insertion using light microscopy. Watkins et al. reported the tendon maturing scoring system to quantitatively evaluate the regenerated tendon [16]. Six histologic parameters, including cellularity, fibrocytes, vascularity, fiber diameter, parallel cells, and parallel fibers, were evaluated to identify the characteristics of the maturity of cellular and intercellular constituents. The sections were stained by the picrosirius red method with 0.2% phosphomolybdic acid hydrate, 0.4% direct red 80, and 1.3% 2,4,6-trinitrophenol (Polysciences, Inc., Warrington, PA, USA) to evaluate collagen fiber localization and analyzed using Zeiss AxioSkop2 and polarizing microscope (Carl Zeiss, Jena, Germany). Picrosirius red staining shows type I and III collagen fibers as yellow and green, respectively, under the polarizing microscope. The collagen content was calculated as a percentage of the pixels of each tendon–bone interface (green/total pixels or yellow/total pixels) using Adobe Photoshop CC 2015 software (Adobe Systems Incorporated, San Jose, USA).

### Mechanical analysis

**Fig. 2:**
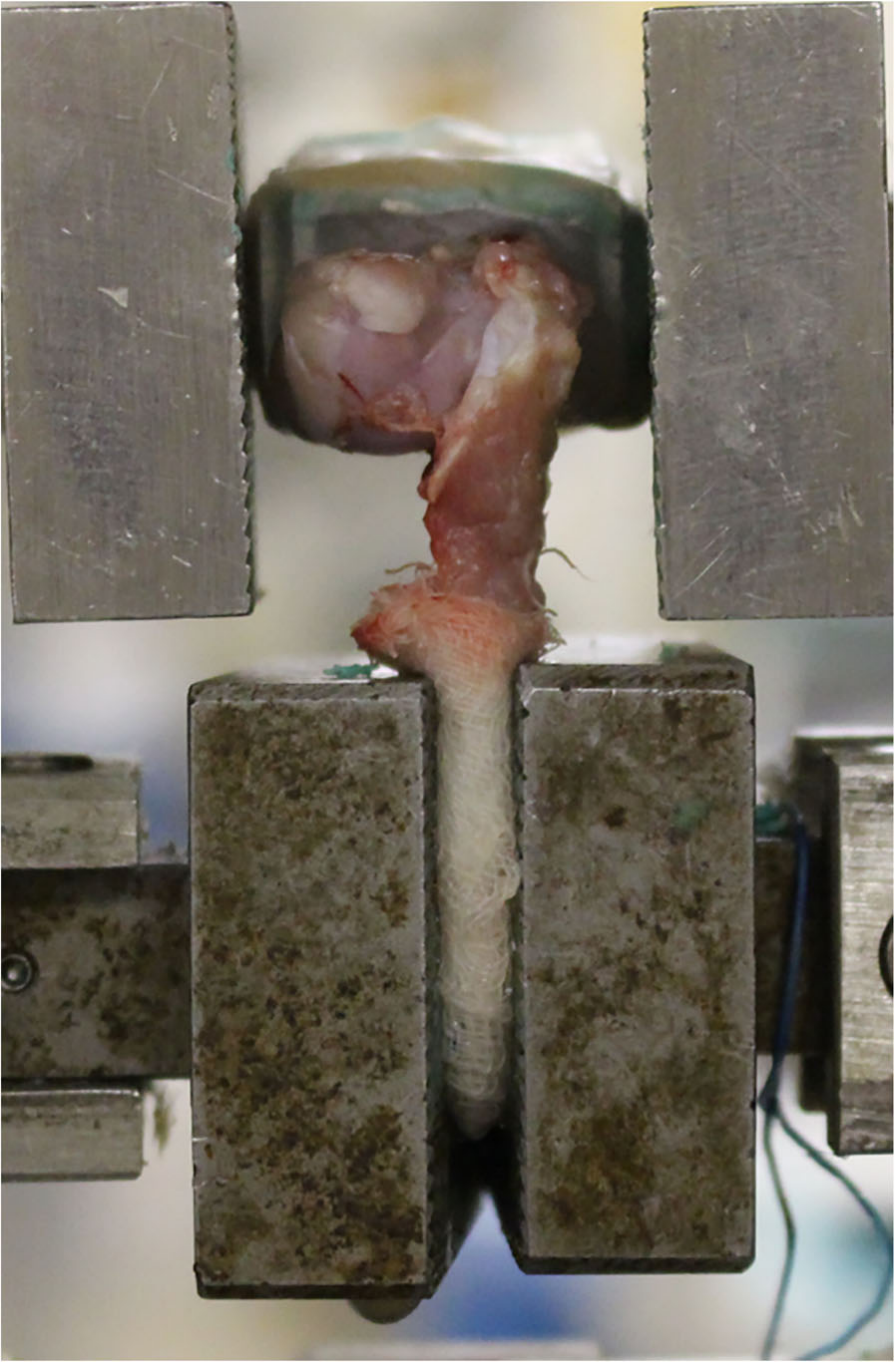
Overview of mechanical analysis with tensile sensor

The infraspinatus tendon–humeral head complexes were harvested from each shoulder, and all soft tissues, except for the infraspinatus tendon, were removed. The humerus was placed in specially designed devices using polymethyl methacrylate resin, the tendon was wrapped with a cotton gauze sponge, and the sponge was sutured to the tendon 10 times using a 1-0 nylon at 5-mm intervals and subsequently clamped to the device [17]. The complex was placed vertically to a tensile sensor (AG-I SHIMAZU Co, Kyoto, Japan) (Fig. 2). Before conduction of the tensile test, the infraspinatus tendon–humeral head complexes were preconditioned with a static preload of 0.5 N for 5 min, followed by 10 cycles of loading and unloading at a strain amplitude of approximately 0.5% at a rate of 20 mm/min. The ultimate failure load was immediately recorded after preconditioning in uniaxial tension at 20 mm/min [18]. The ultimate failure load and stiffness were measured from the load–deformation curve.

### Statistical analysis

Data are presented as the mean ± standard deviation. The Steel-Dwass test was performed to compare the mechanical property and collagen content in the three groups.

Statistical analyses of the tendon maturing score were performed using Student’s *t* test between groups A and R.

*p* values of < 0.05 were considered statistically significant.

## Results

### Histological examination

**Fig. 3:**
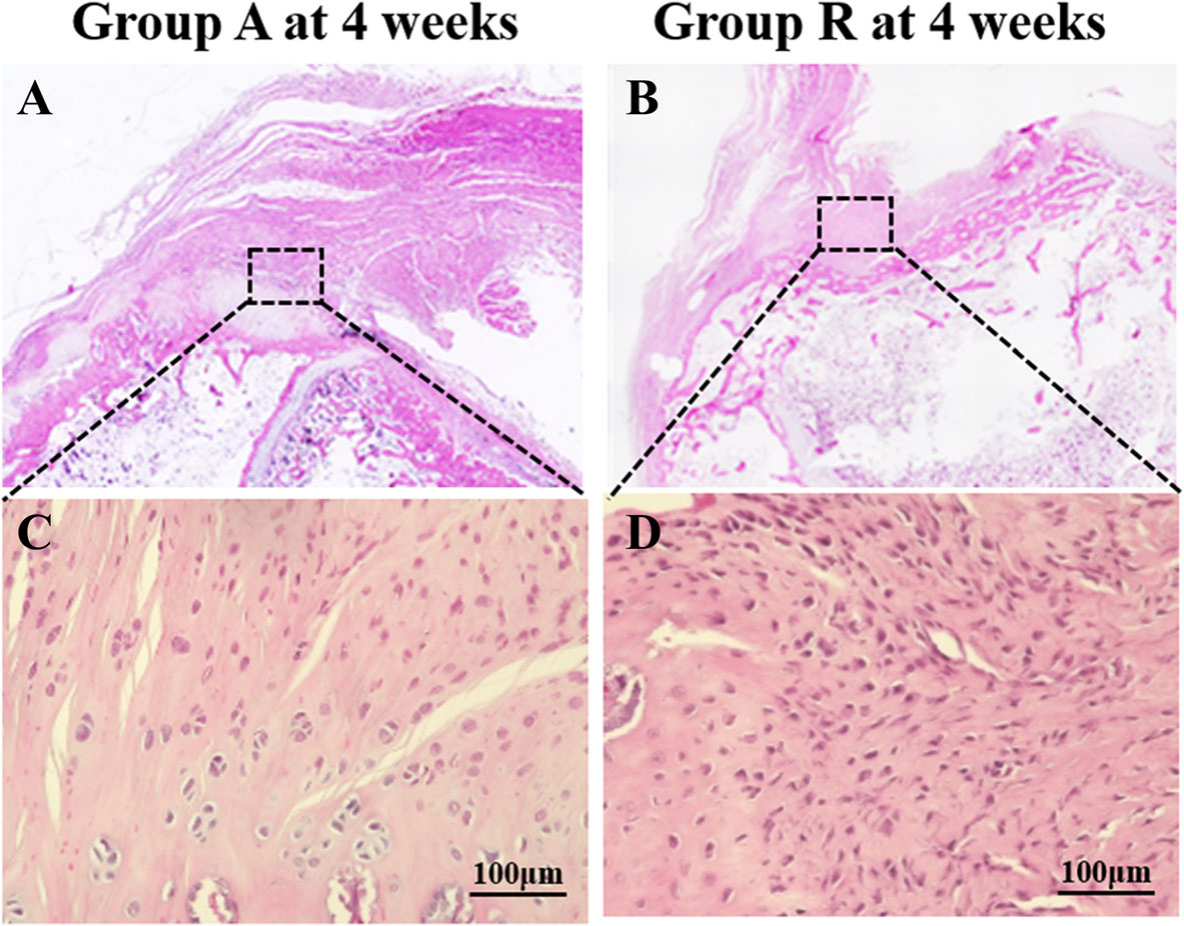
HE staining of treatment groups at 4 weeks postoperatively. Low magnification of **a** groups A and **b** R; high magnification of **c** groups A and **d** R (bar, 100 μm)

In group A, the fibers were thicker and more parallel and cell alignment was more parallel compared with that in group R at 4 weeks postoperatively. A perfect score in the tendon maturing score system is 24 points. At 4 weeks, the tendon maturing scores were 13.8 ± 0.9 and 10.0 ± 1.2 in groups A and R, respectively. A statistically significant difference was found between groups A and R (Fig. 3), (Table 1).

**Table 1:** Results of the tendon maturing score in groups A and R at 4 weeks postoperatively (*p < 0.05)

	Cellularity	Fibrocytes	Vascularity	Fiber diameter	Cells parallel	Fibers parallel	Total
Group A	2.00 ± 0.58	2.00 ± 0.58	2.00 ± 0.58	2.50 ± 0.50*	2.67 ± 0.47*	2.67 ± 0.47*	13.8 ± 0.90*
Group R	1.33 ± 0.47	1.83 ± 0.37	2.00 ± 0.58	1.83 ± 0.37	1.50 ± 0.50	1.50 ± 0.50	10.0 ± 1.15

**Fig. 4:**
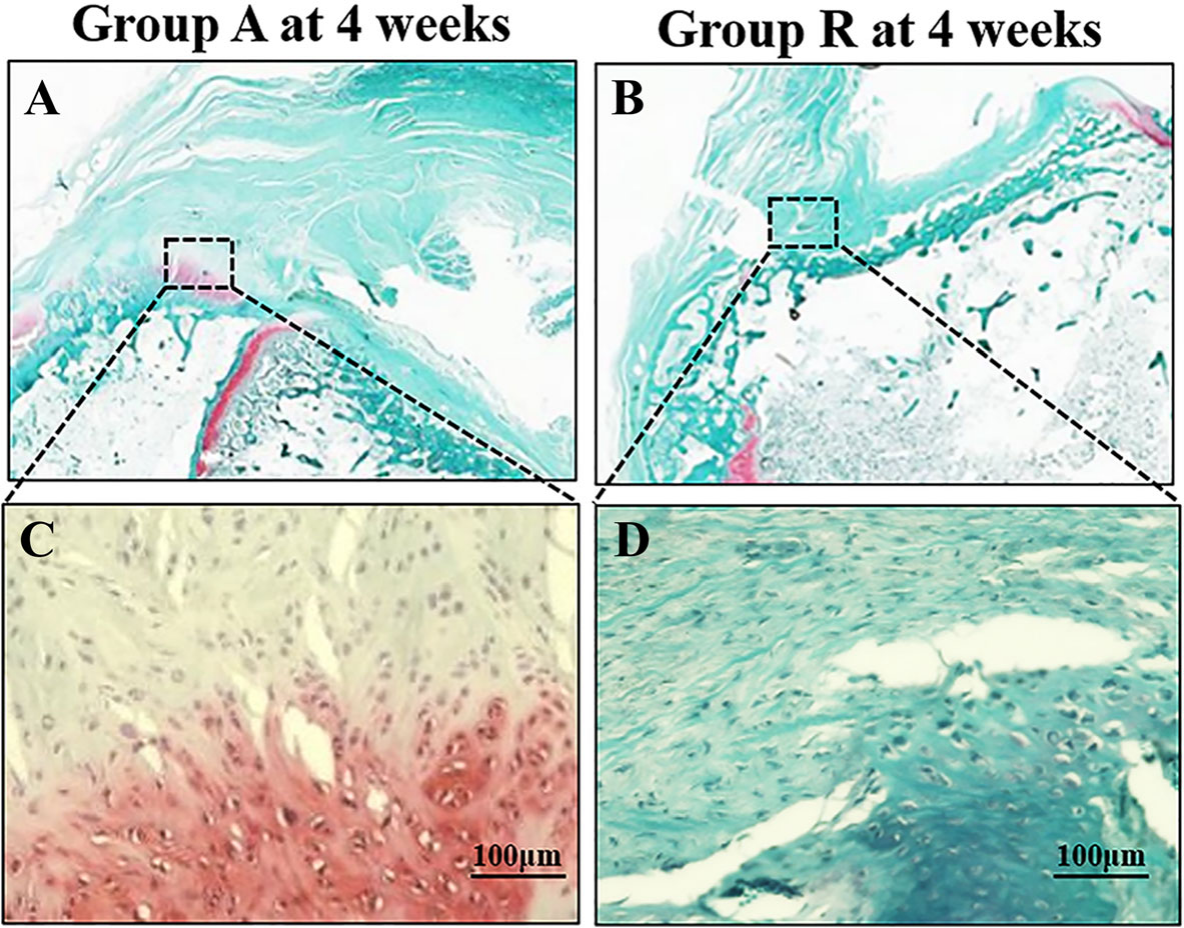
Safranin O staining of the tendon–bone interface of treatment groups at 4 weeks postoperatively. Low magnification of **a** groups A and **b** R; high magnification of **c** groups A and **d** R (bar, 100 μm). White square represents repaired enthesis

In the safranin O staining, proteoglycan staining at the repaired enthesis in group A at 4 weeks postoperatively was stronger than that in group R (Fig. 4).

**Fig. 5:**
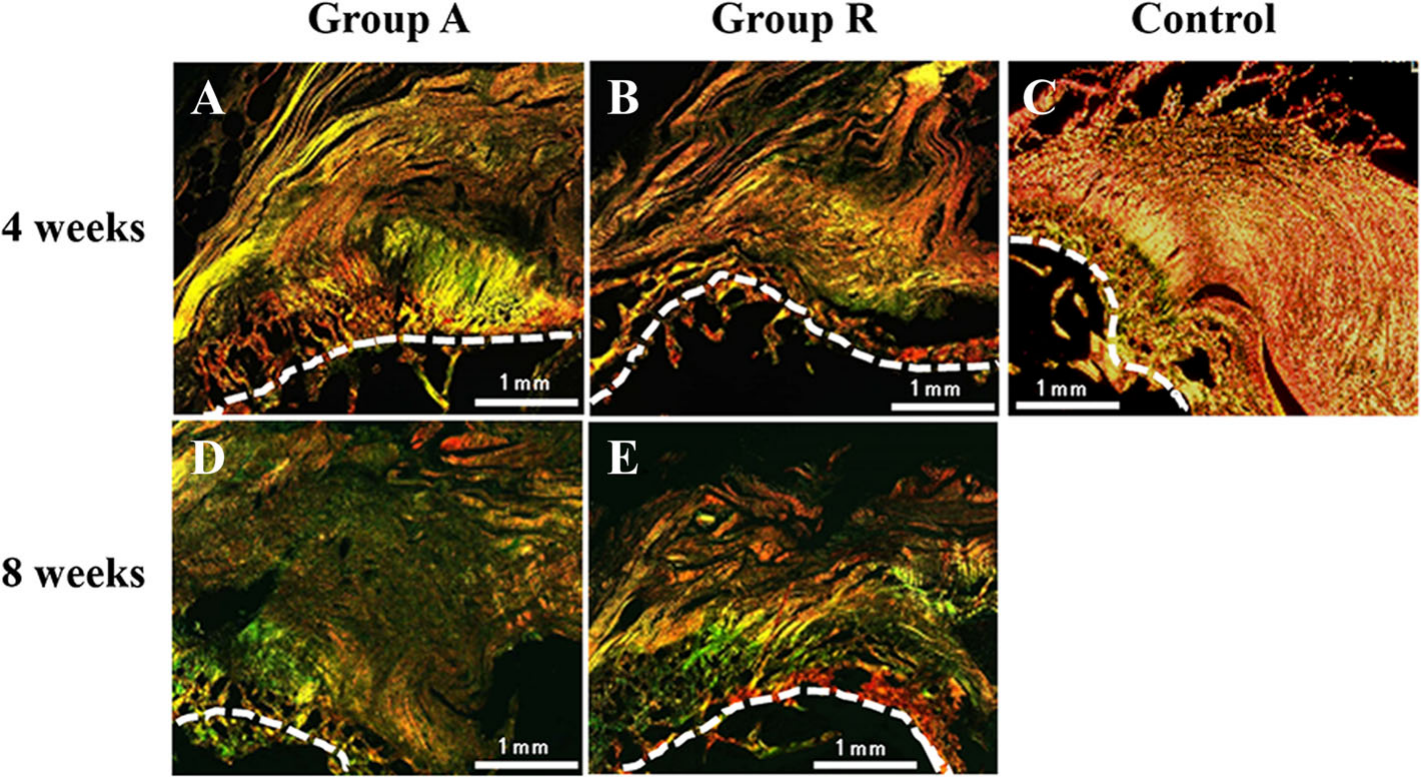
Picrosirius red stain findings at 4 and 8 weeks postoperatively. Picrosirius red staining shows type I and III collagen fibers as yellow and green, respectively. **a** Groups A and **b** R at 4 weeks postoperatively. **c** Control, **d** group A, and **e** group R at 8 weeks postoperatively (bar, 100 μm)

**Fig. 6:**
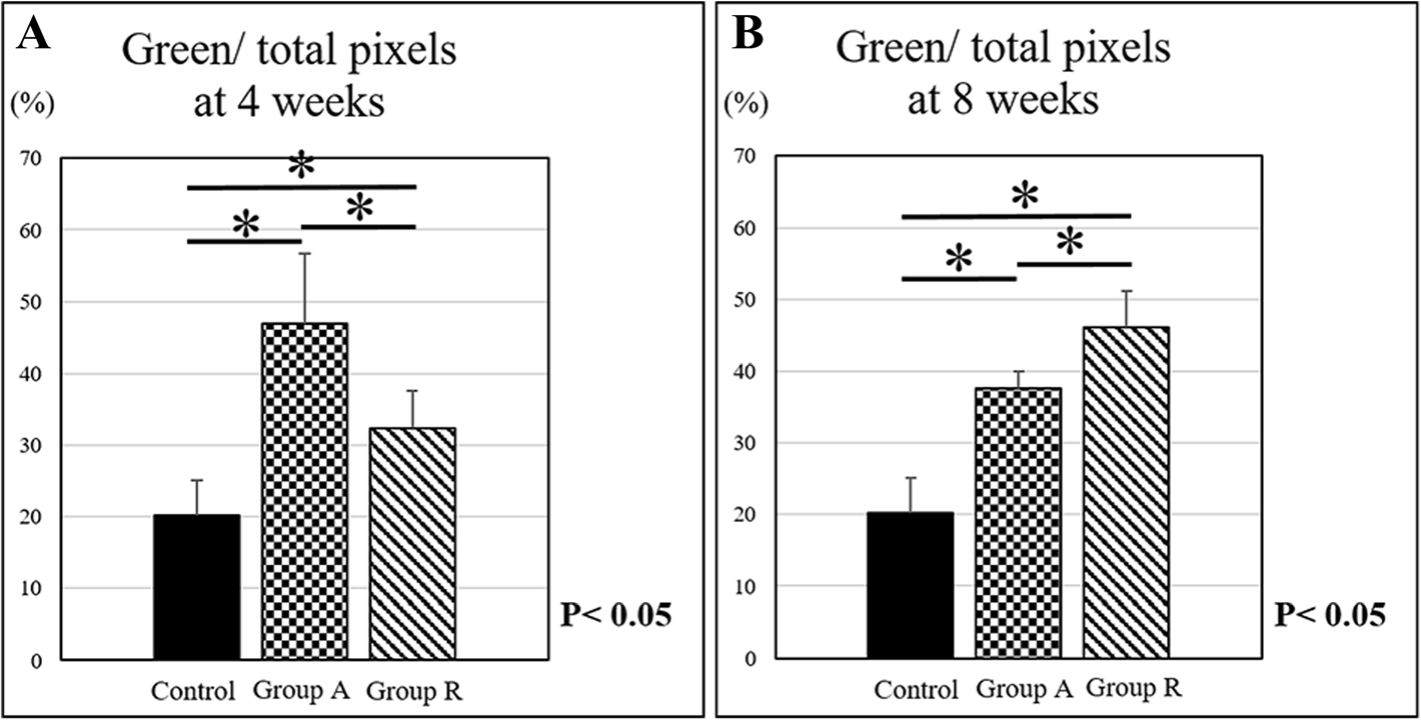
Type III collagen content was calculated as a percentage of the pixels of each image (green/total pixels) in picrosirius red staining at **a** 4 and **b** 8 weeks postoperatively (*p < 0.05)

Picrosirius red staining showed different collagen fiber localizations at the enthesis. Type I and III collagen fibers were observed as yellow and green, respectively, with a polarizing microscope (Fig. 5). Type III collagen in group A (46.9 ± 9.9%) was more strongly expressed than that in group R (32.3 ± 5.3%) at 4 weeks postoperatively. However, the type III collagen expression of group R (46.0 ± 5.0%) was significantly higher than that of group A (37.6 ± 2.4%) at 8 weeks postoperatively. Type III collagen expressions in both operative groups were significantly higher than those in the control group (20.2 ± 4.9%) at 4 and 8 weeks postoperatively (Fig. 6).

**Fig. 7:**
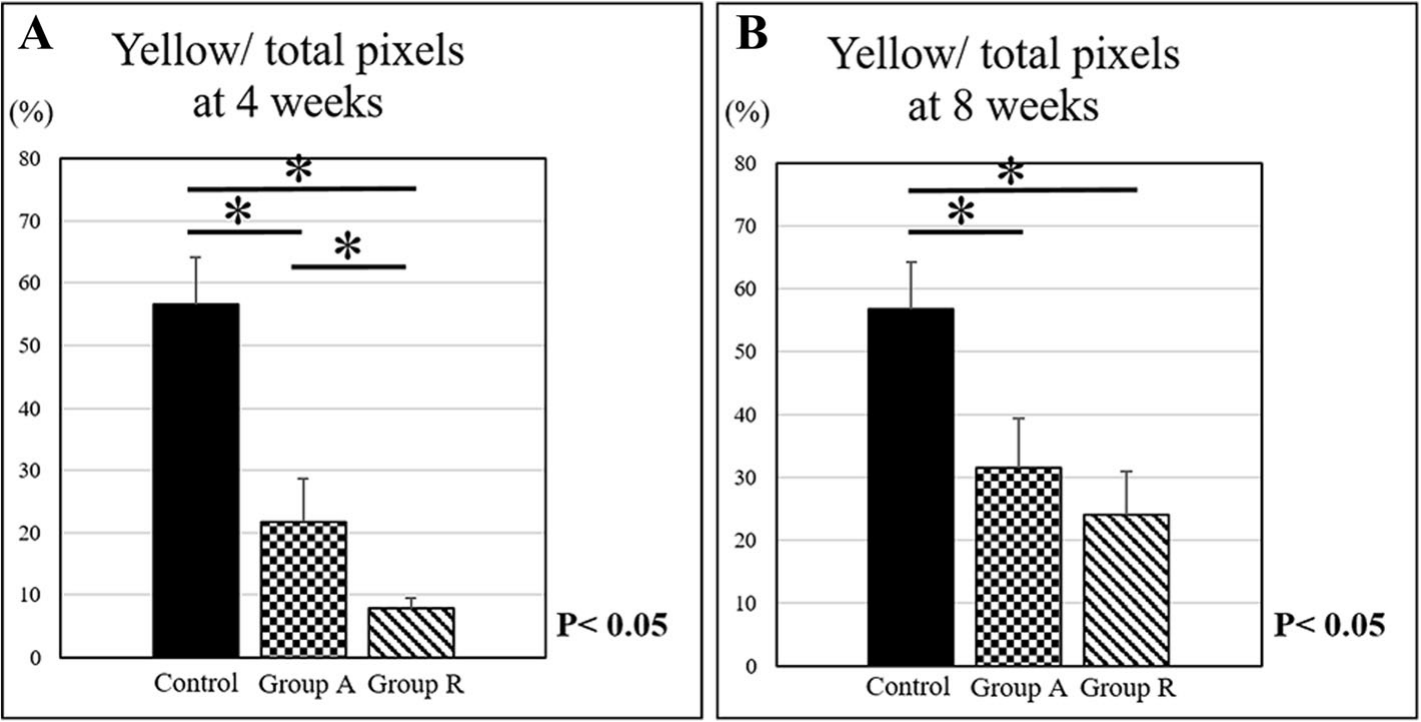
Type I collagen content was calculated as a percentage of the pixels of each image (yellow/total pixels) in picrosirius red staining at **a** 4 and **b** 8 weeks postoperatively (*p < 0.05)

Type I collagen in group A (21.7 ± 7.0%) was more strongly expressed than that in group R (8.0 ± 1.6%) at 4 weeks postoperatively. However, there was no significant difference between group A (31.5 ± 7.7%) and group R (24.0 ± 6.8%) at 8 weeks postoperatively. Type I collagen expression in the control group (56.6 ± 7.6%) was statistically higher than that in both operative groups at 4 and 8 weeks postoperatively (Fig. 7).

### Biomechanical testing

**Fig. 8:**
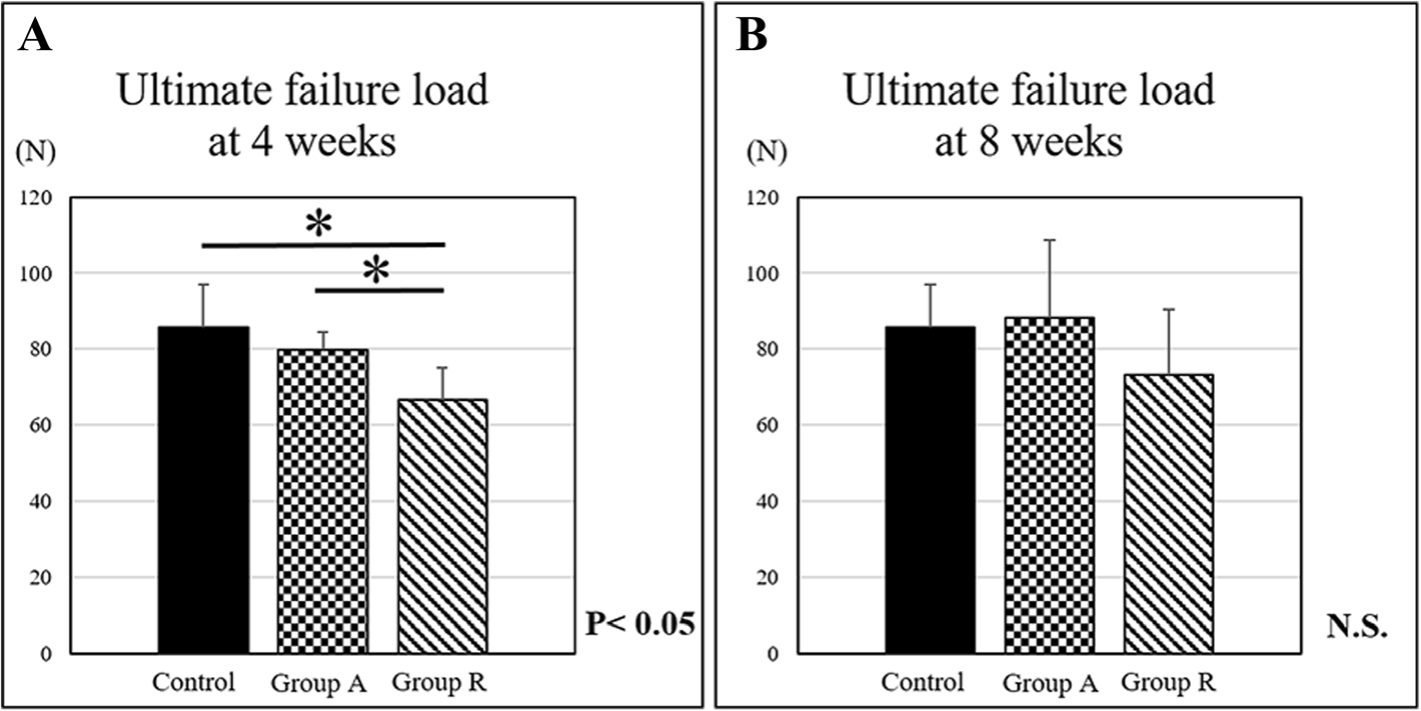
Ultimate failure load of the tendon–humeral head complex at **a** 4 and **b** 8 weeks postoperatively (*p < 0.05)

The ultimate failure load of the tendon–humeral head complex was 79.7 ± 4.9 N and 66.7 ± 8.3 N in groups A and R, respectively, at 4 weeks postoperatively, which showed a statistically significant difference (*p* < 0.05). The failure load of group A at 4 weeks postoperatively was similar to that of the control group (85.6 ± 11.3 N). However, the ultimate failure load of groups A and R was 88.2 ± 20.4 N and 77.3 ± 17.2 N, respectively, at 8 weeks postoperatively, and no significant difference was found among the three groups (Fig. 8).

**Fig. 9:**
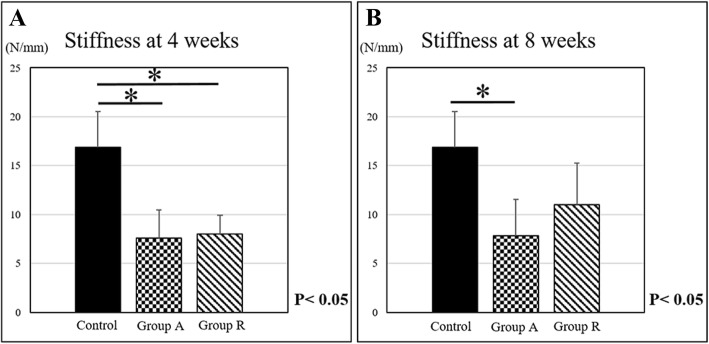
Stiffness of regenerated tissues at **a** 4 and **b** 8 weeks postoperatively (*p < 0.05)

In contrast, the stiffness of groups A and R was 7.6 ± 2.9 N/mm and 7.8 ± 3.7 N/mm, and 8.0 ± 1.9 N/mm and 11.0 ± 4.3 N/mm at 4 and 8 weeks postoperatively, respectively. The stiffness of the control group was 16.9 ± 3.7 N/mm. The stiffness did not show a significant difference between groups A and R, but the stiffness of both groups was significantly lower than that of the control group at 4 weeks postoperatively. A significant difference was observed only between group A and the control group at 8 weeks postoperatively (Fig. 9).

## Discussion

Repair site retear is a common complication of large or massive rotator cuff repairs. Several factors are considered as causes of retear, such as blood circulation disorder due to rotator cuff repair, rotator cuff degeneration, and rotator cuff retraction [12, 19]. Furthermore, when the tear size increases, the traction force applied to the repaired site also increases, leading to weakness of initial fixation force and repaired cuff retear. Alternative therapies, such as tendon transfers, autografts, allografts, and synthetic materials, have been reported to reduce stress with the repair site after repair of massive rotator cuff tears.

We have performed fascia lata augmentation for large or massive rotator cuff tears [15]. In a cadaveric model, rotator cuff repair with augmentation with a fascia lata patch significantly had less gap formation at the tendon–bone interface with cyclic loading compared with non-augmented repair, indicating the possibility of reducing the incidence of rotator cuff repair failure due to addition of the fascia lata patch [20]. In our study, the mechanical strength of fascia lata augmentation was higher than that of the repaired group at 4 weeks postoperatively. Fascia lata augmentation has been suggested to provide the initial fixation strength of the repaired rotator cuff.

The main structural component of the tendon is type I collagen. However, in the early phase of tendon healing, type III collagen increases and gradually replaces type I collagen as the tissue matures. This process is essential for maintaining the structure and function of the tendon [21, 22]. Hirose et al. found that a similar healing process occurs during the healing of rotator cuff tears. In the early phase of healing, repair tissue predominantly produces type III collagen, and type I collagen subsequently increases and replaces type III collagen [23].

In a rat anterior cruciate ligament repair model, Mifune et al. showed that an increase in cellularity and angiogenesis was observed in augmented grafts compared with conventionally reconstructed grafts. Rat-specific type III collagen expression and biomechanical strength in augmented grafts were also significantly higher than that in the conventional reconstruction group [24].

In the present study, picrosirius red staining was used to evaluate collagen fiber localization. In this method, the color of the collagen fiber changes depending on its thickness. As fiber thickness increases, the color changes from green to yellow to orange to red [25]. Because type III collagen fibers are usually thinner than type I collagen fibers, type I and III collagen fibers are stained yellow and green, respectively. Picrosirius red staining revealed type I and III collagen expression in the enthesis. Type III collagen expression in the fascia lata augmentation group at 4 weeks postoperatively was higher than that in the reattachment group. The expression of type III collagen rapidly decreased in augmentation group at 8 weeks. In contrast, type I collagen expression in augmentation group at 4 weeks was higher than that in reattachment group.

Tendon maturation score of augmentation group was higher than that of reattachment group. These results suggested that fascia lata augmentation could stimulate type III collagen expression and type I collagen replacement and promote enthesis healing process in early phase. However, type I collagen expressions of both operative group at 4 and 8 weeks were lower than that of control group. The less expression of type I collagen in the operative group caused lower stiffness compared to the normal tendon.

Strong proteoglycan staining at the enthesis in the fascia lata augmentation group was observed, whereas less staining was observed in the control group in safranin O staining. Proteoglycan is a cartilage matrix produced by chondrocytes. Increased proteoglycan at the repaired enthesis has been reported to lead to increased chondrocytes at the enthesis [26]. The anatomical structure of the enthesis consists of fibrocartilage with the following four zones: ligament substance, unmineralized fibrocartilage, mineralized fibrocartilage, and bone. Because the material properties of the special insertion zone are intermediate between the ligament and bone, a new cartilage transmits loads and decreases stress concentration at the attachment site [18]. Leung et al. reported that new cartilage formation during the healing process was associated with the mechanical property of the tendon–bone interface [27]. Fascia lata augmentation might promote fibrocartilage regeneration at the enthesis and contribute superior mechanical strength compared with the repair without augmentation.

Another possibility of the effectivity of fascia lata graft is its function as a scaffold. Decortication of the footprint was performed to stimulate the bone marrow after rotator cuff repair. Bone marrow stimulation is caused by the migration of bone marrow-derived mesenchymal stem cells (MSCs) [28]. The presence of MSCs in the bone marrow provides a potential for differentiation into tendon tissues [29]. We speculate that the fascia lata autograft over the repaired site might prevent MSCs to spread from the footprint.

The present study has several limitations. First, this rabbit model was an acute rotator cuff injury model. The animal models may differ from chronic human rotator cuff injury. Second, the anatomy between the rabbit shoulder and that of humans are different, and the short rotator cuff muscles of rabbits do not form a rotator cuff that is similar to humans. Third, because rabbits have a greater healing capacity than humans, the tendon–bone healing process in the rabbits progressed faster than that in humans.

In clinical situations, some problems such as hematoma, discomfort, and pain of donor site might be caused after graft harvest. However, Mihata et al. reported that they made autograft for arthroscopic superior capsule reconstruction (SCR) from fascia lata because the tissue is stiff enough to obtain superior shoulder stability after SCR, and no patients had any dysfunction with the harvest site at the final follow-up [30]. Furthermore, autograft has no concern of an immune reaction, zoonosis, or foreign body reaction [15].

The results from the present study suggest that fascia lata augmentation could stimulate biological healing and provide initial fixation strength of the repaired rotator cuff.

## Conclusion

The fascia lata augmentation for massive rotator cuff tears could stimulate type III collagen expression and type I collagen replacement and promote enthesis healing process in early phase. Mechanically, the fascia lata augmentation provided initial fixation strength of the repaired rotator cuff.

## Abbreviations

MSCs: Bone marrow-derived mesenchymal stem cells
HE: Hematoxylin–eosin
ISP: Infraspinatus
SCR: Superior capsule reconstruction
